# Online mindfulness meditation for mild cognitive impairment and mild dementia: A feasibility study protocol

**DOI:** 10.1371/journal.pone.0336583

**Published:** 2025-11-25

**Authors:** Jiuhong You, Zunera Khan, Reena Swaroop, Yan Sun, Latha Velayudhan, Dag Aarsland

**Affiliations:** 1 Centre for Healthy Brain Ageing, Psychological Medicine, Institute of Psychiatry, Psychology & Neuroscience, King’s College London, London, United Kingdom; 2 South London and Maudsley NHS Foundation Trust, London, United Kingdom; 3 School of Rehabilitation Medicine, Henan University of Chinese Medicine, Zhengzhou, Henan, China; 4 Centre for Age-Related Medicine, Stavanger University Hospital, Stavanger, Norway; UofM: The University of Memphis, UNITED STATES OF AMERICA

## Abstract

**Introduction:**

Mild cognitive impairment (MCI) is an intermediate stage between normal aging and mild dementia. Patients with MCI and dementia usually experience impairment in cognitive functions such as memory, executive function, and processing speed. They may also develop neuropsychiatric symptoms, such as depression, anxiety, and agitation. While previous studies suggest that mindfulness meditation may benefit this population, the feasibility of delivering such interventions online remains unclear. Therefore, this study aims to investigate the feasibility and acceptability of an eight-week, online-delivered mindfulness program for people with MCI and mild dementia.

**Methods:**

This study will recruit 32 participants over 60 years old with MCI or mild dementia in the UK. Participants will attend a weekly live online mindfulness meditation session, led by an experienced mindfulness teacher for eight weeks. Each session lasts 2.5 hours. In addition, participants will be encouraged to do daily home practice.

The primary outcomes are feasibility and acceptability of an online program, assessed through participation records and semi-structured interviews. Secondary outcomes include participants' changes in cognitive function, mood, sleep, quality of life, mindfulness, and resilience.

**Discussion:**

Mindfulness meditation delivered online could help reduce travel burdens and overall costs. This study aims to assess the usability and potential effects of online-delivered programs for this population, providing evidence to support the use of remote interventions in the care of older adults with cognitive impairments.

**Study registration:**

ClinicalTrials.gov Identifier: NCT06768450.

## 1 Introduction

Mild Cognitive Impairment (MCI) represents an intermediate stage between normal aging and early dementia, affecting memory, executive functions, processing speed, and reasoning [1]. Its prevalence for individuals over 65 years old is about 3% to 22% [2]. In addition, 35–85% of these MCI patients have neuropsychiatric symptoms, including depression, irritability, apathy, anxiety, agitation, and sleep disturbances. These symptoms often co-occur, and may have a mutual or cumulative effect on disease progression [3]. Notably, individuals with MCI are at an increased risk of developing dementia, with an estimated10–12% of MCI patients progressing to Alzheimer’s disease every year [4].

Therefore, implementing effective preventive interventions to slow cognitive decline before the onset of dementia symptoms is critical [5]. If the onset and progression of dementia could be delayed by just one year through any form of intervention, there will be approximately 9.2 million fewer cases of dementia in 2050 [6].

Meditation, encompassing a variety of emotional and attentional regulatory training exercises [7], has demonstrated benefits in improving cognition, well-being, and health in older adults, potentially offsetting cognitive decline or delaying the onset of dementia [8]. Meditation training programs typically include sessions led by an instructor, and daily home practice. Various meditation practices exist, with mindfulness-based stress reduction (MBSR) being one of the most studied. Developed in the 1970s by Jon Kabat-Zinn [9], MBSR is an 8-week program that includes weekly group meetings and guidance for daily meditative practice, culminating in a day-long meditation retreat. Some studies suggest that MBSR may enhance attention, executive functioning, or working memory in healthy middle-aged or older adults [10–13].

The benefits of mindfulness meditation were also observed in mindfulness itself [14–16], coping strategies [15], quality of life [17,18], depression [17,19], anxiety [17], cognitive function [14,19–21], including executive function [10], and verbal memory [22]. A meta-analysis found that mind-body interventions including MBSR, improved cognitive function, everyday activities functioning, memory, resilience, and mindfulness in older adults with MCI [12].

Most mindfulness studies have been conducted in face-to-face setting, posing economic burdens related to transportation and presenting challenges for patients unable to travel. During the COVID-19 pandemic, online psychological interventions were particularly significant for improving patients’ psychological and physical well-being [23]. Digital interventions offer a cost-effective and accessible means for fostering positive behaviour change [24]. Previous studies have demonstrated that social media-based mindfulness-based interventions are feasible and acceptable [25], with minimal difference in effectiveness compared to in-person delivery [26].

Telemedicine has also emerged as a valuable alternative to in-person clinical visits for patients with dementia. A systematic review found that individuals with dementia reported high levels of convenience, satisfaction, comfort, and a willingness to recommend telemedicine to others living with the same condition [27]. However, several challenges must be considered when delivering online mindfulness meditation programs to older adults with cognitive impairments. They may experience hearing loss and vision impairment, reduced fine motor coordination, and cognitive decline [28]. These difficulties might be worsened in the context of telemedicine interactions. In addition, to the best of our knowledge, no previous studies have investigated the feasibility of online group-based mindfulness meditation programs for older adults with MCI and dementia. Therefore, our study aims to employ a mixed-methods approach to investigate the feasibility, acceptability, and preliminary effects of online 8-week group-based mindfulness intervention among older adults with MCI and mild dementia. Here we describe the design, recruitment strategy, eligibility criteria, the intervention and the outcome measurements.

## 2 Materials and methods

### 2.1 Study aims

- **Primary aim**

The primary aim of the present study is to investigate the feasibility and acceptability of an online delivered 8-week mindfulness meditation program for older adults with MCI and mild dementia.

- **Secondary aim**

The secondary aim of this study is to provide preliminary estimates of the potential effects of online mindfulness meditation on cognitive function, mood, mindfulness, and quality of life in patients with MCI and mild dementia, to inform the design of a future randomized controlled trial, rather than formally evaluating treatment efficacy.

### 2.2 Eligibility criteria

- **Inclusion criteria**
  - More than 60 years old
  - MCI: MCI is based on subjective and objective evidence of cognitive decline, with relatively preserved daily function as described in National Institute on Aging and Alzheimer’s Association (NIA-AA) diagnostic guidelines [29]
  - Mild dementia: As described in NIA-AA research framework [30], mild dementia is defined as substantial progressive cognitive impairment affecting several domains and/or neurobehavioral disturbance. It can be documented by the individual’s or observer's (e.g., study partner) report, or by changes on longitudinal cognitive testing. There is a clearly evident functional impact on daily life, affecting mainly instrumental activities. Individuals are no longer fully independent and may require occasional assistance with daily activities
  - Access to an electronic device (smartphone/computer/tablet) and the internet
  - Be able and willing to give informed consent to participate by signing the Consent Form
- **Exclusion criteria**
  - Current active or past significant experience with meditation
  - Clinically significant mental or physical health disorders beyond MCI or dementia, that can impact cognition and/ or ability to complete the study (e.g., severe depression, suicidality, psychosis, post-traumatic stress disorder, social anxiety (difficulty with being in a classroom situation), or other major psychiatric diagnosis, severe impairments in eyesight, hearing or upper limb motor movements
  - Inability to commit to attending classes (if someone is likely to miss three or more classes)
  - Currently involved in any other interventional research
  - Moderate or severe dementia

### 2.3 Outcomes

- **Primary outcomes**

The primary outcomes include descriptive data on recruitment, participation, retention, attrition, and compliance, which will be recorded by a researcher during the recruitment process and mindfulness training. The satisfaction will be assessed via client satisfaction questionnaire (CSQ-8). Semi-structured interviews will be conducted after the intervention to explore participants’ experiences.

- **Recruitment rate**: The recruitment rate will be calculated as the following:

$Recruitmentrate(\%)=\frac{Numberofparticipantsenrolled}{Numberofpotentialparticipantscontacted}*\text{100}$

The reasons why potential participants could not attend (e.g., technology limitations) will be recorded.

- **Participation rate**: Program participation refers to enrolled subjects who completed at least 50% of the planned practice sessions during the study period, which is defined as attending more than four sessions.

$Participation\;rate(\%)=\frac{number\;of\;participants\;who\;attended\;more\;than\;\text{4}\;sessions}{number\;of\;participants\;initially\;enrolled}*\text{1 0 0}$

A participation rate exceeding 60% indicates that the study is feasible.

- **Retention rate**:

$$Retention\;rate(\%)=\;\frac{number\;of\;participants\;who\;completed\;the\;study}{number\;of\;participants\;initially\;enrolled}*\text{1 0 0}$$

- **Attrition rate**:

$$Attrition\;rate\;(\%)=\frac{number\;of\;participants\;who\;dropped\;out}{number\;of\;participants\;initially\;enrolled}*\text{1 0 0}$$

Their timing and reasons for withdrawals will also be recorded.

- **Compliance**:

Compliance will be assessed in two ways. Self-rated adherence will be captured through two questions about live sessions and homework assignments. Participants will be asked “Over the past eight weeks of **live sessions**, how often did you follow the meditation instructions as required?” and “Over the past eight weeks of **homework**, how often did you follow the meditation steps (e.g., duration, frequency, and method)?” Participants will respond using a 5-point Likert scale [31]: 1 = Never, 2 = Rarely, 3 = Sometimes, 4 = Often, 5 = Alway

Objective compliance will be evaluated through class attendance, and record in participants’ at-home practice workbooks. Both subjective and objective compliance outcome will be reported descriptively.

The above data will be recorded by the research staff during recruitment and throughout the 8-week mindfulness training. Weekly course attendance and adverse events will be documented, and 4-week midpoint feasibility outcomes will be reported to detect early disengagement.

- **Client satisfaction questionnaire (CSQ-8):** The CSQ-8 [32] is a standardized 8-item tool that has demonstrated good reliability and validity in mental health services. Items are scored from 1 (low satisfaction) to 4 (high satisfaction) with different descriptors for each response point. Total scores range from 8 to 32, with higher scores indicating greater satisfaction. Example questions include “If a friend were in need of similar help, would you recommend the program to him or her?” and “If you were to seek help again, would you come back to the service?”
- **Adverse events:** Adverse events during the study will be recorded in terms of patient characteristics, nature of the event, date, outcome, action taken, and follow-up details.
- **Qualitative data collection**

**Semi-structured interviews** with participants and study partners will be conducted by a study researcher within 4 weeks after completion of the meditation intervention, via telephone or video conferencing. Each individual interview will be less than an hour.

The aim of the interview is to further explore participants’ experiences with the program, including their overall feeling, specific benefits and challenges, the support they need, and their plans for future practice. Each participant may identify a study partner—this could be a spouse, friend, child, or another individual who knows them well. The study partner should be able to remind the participant to engage in the practice and assist with any technology-related issues. The experiences of study partners and their supportive roles will also be examined.

Interviews will be audio/video recorded with participants consent and the recordings will be transcribed. An inductive thematic analysis will be completed, supported by Nvivo software [33].

In addition, the session audio will be recorded, and homework reflections, group discussions, and chat box input during sessions will be collected to examine participants’ experiences, changes, and difficulties.

- **Secondary Outcomes**

Secondary outcomes include cognitive testing and questionnaires assessing sleep, mood, mindfulness, quality of life, instrumental activities of daily living, and resilience. These assessments will be conducted before and after the 8-week program using Qualtrics. Participants can either click the link sent by email to complete the questions independently, or they can ask for researcher’s assistance via telephone and/or video conferencing (e.g., MS Teams). The process may take approximately 50 minutes in total.

- **Cognitive function** will be assessed with the Modified Telephone Interview for Cognitive Status (TICS-M)

The TICS-M is a brief, 13-item test of cognitive functioning with scores ranging from 0 to 50. Questions related to orientation, repetition, naming, and calculations are covered. Additionally, a 10-item non-semantically related word list is recalled both immediately and after a delay of about 5 minutes filled with distractor questions. Past research has demonstrated that the TICS-M is as reliable and valid as face-to-face administration, with a sensitivity of 94% and a specificity of 100% for distinguishing normal controls from individuals with dementia [34].

- **Anxiety and depression** will be assessed with Hospital Anxiety and Depression scale (HADS)

The HADS [35] is used in general medical hospital outpatient clinics to assess patients’ mental health. It consists of two subscales (anxiety and depression), each comprising seven items scored from 0 to 3, with a maximum total score of 21 per subscale. A score of 0–7 on either subscale is considered within the normal range, a score of 11 or higher indicates a probable mood disorder, and a score of 8–10 suggests the possible presence of the respective condition.

- **Sleep quality** will be assessed with Pittsburgh sleep quality index (PSQI)

The PSQI is a self-rated questionnaire designed to assess sleep quality and disturbances over a 1-month period [36]. Nineteen individual items generate seven component scores: subjective sleep quality, sleep latency, sleep duration, habitual sleep efficiency, sleep disturbances, use of sleeping medication, and daytime dysfunction. A global PSQI score greater than 5 has been shown a diagnostic sensitivity of 89.6% and specificity of 86.5% (κ = 0.75, *P* < 0.001) in distinguishing good and poor sleepers. The clinimetric and clinical properties of the PSQI support its utility both in psychiatric clinical practice and research.

- **Quality of life** will be assessed with EQ-5D

The 5-level EQ-5D version (EQ-5D-5L) [37] was introduced by the EuroQol Group in 2009, which comprises five dimensions: mobility, self-care, usual activities, pain/discomfort and anxiety/depression. Each dimension has 5 levels: no problems, slight problems, moderate problems, severe problems, and extreme problems. The second part of the questionnaire is the EQ VAS, so called because it incorporates a Visual Analogue Scale. This captures the respondent’s overall assessment of their health on a scale from 0 (worst health imaginable) to 100 (best health imaginable). The EQ-5D has been widely used in the dementia population and exhibited convergent validity with other dementia-related outcomes [38].

- **Instrumental activities of daily living (IADL)** will be assessed with a short version of the Amsterdam IADL Questionnaire (A-IADL-Q-SV).

The A-IADL-Q-SV [39] was developed from original A-IADL-Q, which is shortened to 30 items from 70 items, resulting in an administration time of 10–15 minutes. The A-IADL-Q-SV will be completed by a study partner. And it has maintained the psychometric quality of the original version including household, administration, work, computer use, leisure time, appliances, transport, and other activities; and presented a high internal consistency (0.98).

- **Mindfulness** will be assessed with the five-facet mindfulness questionnaire (FFMQ).

The FFMQ [40] is a self-help and self-scorable measurement on the five aspects of mindfulness namely: observation, description, acting with awareness, non-judgmental inner experience, and non-reactivity. The test consists of 39 items that measure the five facets, and the scores provide an estimate of where we stand in terms of mindfulness and self-awareness. Construct validity has been demonstrated [41].

- **Resilience** will be assessed with the Connor-Davidson Resilience Scale 10 (CD-RISC 10)

The CD-RISC 10 [42] is a 10-item test to measure resilience or how well one is equipped to bounce back after stressful events. The 10-items are scored on a 5-point scale ranging from 0 (not true at all) to 4 (true nearly all the time). Cosco et al. [43] concluded that the CD-RISC potentially demonstrates sufficiently acceptable psychometric properties in older populations.

- Demographic data

Demographic information will be collected on participants’ age, gender, education level, previous profession, stage of cognitive impairment or dementia diagnosis, ethnicity, marital and civil status, employment status, health conditions, and medication use.

- Study design

This is a feasibility study with mixed method qualitative–quantitative design. The qualitative component focuses on exploring participants’ and study partners’ experiences of the 8-week mindfulness program through interviews. The quantitative component assesses objective outcomes, including retention rate, mood, and sleep quality scales. By integrating these two methods, this study aims to provide a comprehensive understanding of the feasibility and acceptability of an online 8-week mindfulness meditation program for older adults with MCI and mild dementia.

### 2.4 Statistical analyses

The proposed sample size for participant recruitment is 32, as this number is considered acceptable for a feasibility study [44]. Sophie [45] concluded that while a sample size justification is important for pilot and feasibility trials, a formal sample size calculation may not always be appropriate. Their analysis of pilot and feasibility studies registered in the UKCRN database found a median sample size of 30. Browne [46] suggested that a general rule of thumb is to recruit 30 or more participants to estimate a parameter. Based on this evidence and considering eight participants per group, 32 is considered an appropriate sample size for this study.

As suggested, the analysis of feasibility studies should mainly be descriptive [47]. Therefore, primary outcome measures will be summarised using descriptive statistics, including proportions, means, ranges, and standard deviations (SDs) where appropriate (e.g., recruitment, retention, attrition, and compliance). Secondary outcomes (e.g., sleep quality, cognitive function) will similarly be reported as before-and-after means, SDs, mean differences, and 95% confidence intervals. These analyses will not be powered to detect intervention efficacy. Instead, the findings will be used to identify practical challenges, refine study procedures, and inform the design of a future randomised controlled trial.

For missing data, we will report them descriptively as attrition rates and use qualitative methods to investigate why participants drop out.

The qualitative data will be analysed using Braun and Clarke’s six phase method [48] for conducting a thematic analysis. The transcripts will be coded by two researchers independently. From the codes, categories will be identified independently and then discussed to reach consensus. Themes across the dataset will be checked by a researcher with qualitative experience who is independent of the initial analysis to ensure that no aspect of the data has been under or overrepresented. Themes will be reported to inform any amendments or improvements to the intervention.

### 2.5 Study registration and ethical considerations

This study has been registered at ClinicalTrials.gov under the identifier NCT06768450.

The study will be conducted in accordance with the declaration of Helsinki [49]. Participants will be given at least 48 hours to review the study information before providing written informed consent. Only participants with capacity to consent will be included. This research has received ethics approval from London Bridge Research Ethics Committee, with REC reference: 25/LO/0304.

## 3 Study procedures

### 3.1 Recruitment Platforms

In this single-centre study, the subjects will be recruited from the South London and Maudsley Hospital (SLAM) NHS trust remote Brain Health Clinic [50] and other SLAM memory clinics, and Join Dementia Research (https://www.joindementiaresearch.nihr.ac.uk/). Recruitment materials, such as study information and posters, will also be shared via social media platforms, as well as newsletters and circular emails from King’s Health Partners. Informed consent from participants will be obtained before the intervention.

### 3.2 Interventions

The mindfulness meditation modified from Breathwork Mindfulness for Stress program, is derived from MBSR and Mindfulness Based Cognitive Therapy (MBCT) [51]. However, it uses shorter meditations to accommodate individuals who cannot sit for 40 minutes [52]. The mindfulness meditation program encourages greater self-acceptance, kindness and compassion to self, and builds on this concept by encouraging people to find pleasure in life despite suffering and encourages kindness and compassion towards others, and to increase their social connections in order to counteract the social isolation common in people with chronic and long-term illness [52].The program will be led by a well-trained Breathwork mindfulness instructor, conducted remotely via MS Teams once a week for 8 weeks, with each session lasting 2 ~ 2.5 hours consisting of body scan, mindful movement, various meditations, with different ideas every week (the details are shown in **Table 1**). Given the absence of prior online meditation studies specifically targeting individuals with MCI and dementia, we have derived our group size estimate from face-to-face studies, which typically range between 6–12 participants [22,53,54]. Considering that interaction may be reduced in virtual [55], we have opted to limit each group to 8 participants to ensure the efficacy and quality of the online training sessions. To better fit the need of patients with MCI and mild dementia, the eight-hour silent retreat will be removed, with the homework highly encouraged but not compulsory.

**Table 1:** The mindfulness meditation courses for people with MCI and mild dementia.

	Time locations	Home practice
Week 1 **Learning to choose** (2.5 hours)	Welcome and group agreement (10 mins) Introductions to each other (20 mins) Intro to the course and this week’s idea (10 mins) What is mindfulness? An activity, e.g., Raisin exercise (20 mins) Mindfulness ideas: reacting and responding, primary and secondary experience (20 mins) Break: free to chat with fellow participants (20 mins) Meditation: body scan practice and enquiry (30 mins) Closing and homework (20 mins)	**Meditation**: practice a body scan twice a day **Mindfulness in action**: do one routine activity mindfully every day (page 33–34 in the workbook) **Reading:** chapters 1 and 2 of the workbook
Week 2 **Coming to our Senses** (2.5 hours)	Body Scan (20 mins) Last week Review using break out room discussion (30 mins) Recap and intro to this week’s idea (10 mins) Idea: doing and being modes of mind (15 mins) Break (20 mins) Posture workshop (10 mins) Intro to Mindfulness of Breathing and enquiry (30 mins) Closing and homework (15 mins)	**Meditation**: Practice Mindfulness of Breathing once a day **Meditation posture:** Find a sitting posture that you can keep fairly comfortably for 10 minutes (Pages 33–36 of Workbook) **Mindfulness in Action**: Do a few things more slowly than usual (Page 53–54 in the Workbook) **Reading:** Chapters 3 of The Workbook
Week 3 **Working with Thoughts** (2.5 hours)	Intro to mindful movement (25 mins) Last week Review in groups (30 mins) Recap and intro to this week’s idea (10 mins) Intro to 3 step breathing space and enquiry (10 mins) Break (20 mins) Intro to mindfulness of thoughts (25 mins) Mindfulness of breathing – letting go of thoughts and enquiry (20 mins) Closing and homework (15 mins)	**Meditation** Continue with the **Mindfulness of Breathing** once each day **Mindful movement**: once each day (instructions and illustrations on Pages 33–36 in the Workbook) **Specific meditations**: Listening to sounds and Mindfulness of breathing – working with thoughts (twice through the week) Three Step Breathing Space **Mindfulness in Action**: “Take a break” (Page 85–87 in the Workbook) **Reading**: Chapters 4 of The Workbook
Week 4 **Overview: Working with Difficult Experiences** (2.5 hours)	Mindfulness of Breathing (20 mins) Last week review in groups (30 mins) Recap and intro to this week’s idea (10 mins) Idea: Acceptance of Difficult Experiences (15 mins) Break (20 mins) Idea: the paradox of mindfulness (10 mins) Acceptance Meditation (20 mins) Mindful Movement (10 mins) Closing and homework (15 mins)	**Meditation** **Body scan** once each day **Mindfulness of Breathing** once each day **Specific meditations**: being with unwanted experience and mindfulness of breathing – working with charged thoughts (twice through the week) **Mindfulness in Action**: Accept a difficult experience (pages 106–107 in the Workbook) **Reading**: Chapters 5 of The Workbook
Week 5 **Noticing the Good Things** (2.5 hours)	Body Scan – especially looking for pleasant sensations and savoring them (20 mins) Last week review in groups (30 mins) Introduction to Idea: Negativity Bias (10 mins) Break (30mins) Seeking out the pleasant exercise (20 mins) Letting in the good practice and enquiry (20 mins) Mindful Movement with music (10 mins) Closing and homework (10 mins)	**Meditation-**Simply practice two meditations each day from the following (Practice Book on page 33): **Body Scan** **Mindfulness of Breathing** **Mindful Movement** **Mindfulness in Action**: Letting in the good (pages 117–119 in the Workbook) **Reading:** Chapters 6 of The Workbook
Week 6 **Kindness** (2.5 hours)	Mindful movement and mindfulness of breathing (25 mins) Last week review in groups (30 mins) Introduction to Idea: The 3 Major Emotion Systems (20 mins) Break Kindness to self: introductory exercises, meditation practice and enquiry (35 mins) Closing and homework (20 mins)	**Meditation:** Kindness to self meditation combined with either body scan, mindfulness of breathing or mindful movement for a longer practice **Mindfulness in Action**: Spend some time in the Green Circle (pages 136–138 in the Workbook) **Reading:** Chapters 7 of The Workbook
Week 7 **The Social Dimension of Mindfulness** (2.5 hours)	Kindness to Self and a friend meditation (20 mins) Last week review in groups (30 mins) The Exhaustion Funnel, Sustainers and Drainers exercise (30 mins) Break (20 mins) Kindness to others exercise (20 mins) Kindness to others meditation and enquiry (20 mins) Closing and homework (10 mins)	**Meditation:** practice **Kindness towards someone you like,** and **Kindness to Everyone** **Mindfulness in Action**: Choose to respond rather than react (pages 152–155 in the Workbook)
Week 8 **Where to go from here** (2.5 hours)	Groups choice of guided meditation (20 mins) Last week review and intro to this week (10 mins) Review of the course (20 mins) Mindful movement and mindfulness of breathing (20 mins) Break Complete Evaluation Form or ‘letter to myself’ (10 mins) Where to go from here (10mins) Goodbye (20 mins)	–

Participants will receive online recordings of guided meditations (body scan, mindfulness for breathing, mindful movement, and specific meditations based on the weekly topic), along with a home practice manual that includes space for them to write notes on their experience.

### 3.3 Data management

All research data will be pseudonymised. Each participant will be assigned a unique numerical ID number to ensure data confidentiality and anonymity. Associations between names of participants/contact details and ID numbers will be detailed within an electronic password protected database and stored on a password protected encrypted secure network. Access to this document will be restricted to the chief investigator and study researchers.

### 3.4 Assessment of safety

There is no foreseen risk to the participants. Some mindful movement practice might ask participants to stand, walk, or do some simple stretching. If a participant hears guidance that they know is not appropriate for their body or condition, or if anything causes pain, they should disregard the teacher and either modify the pose/movement, rest and imagine doing the pose/movement, or notice and acknowledge any thoughts or emotions that may be arising in the experience of not doing the pose/movement.

The teacher offers modifications or adaptations to poses/movements to meet the variety of capacities in the class.

Any adverse events will be recorded immediately during the study, by open-ended questions.

### 3.5 Study timetable

**Fig 1:**
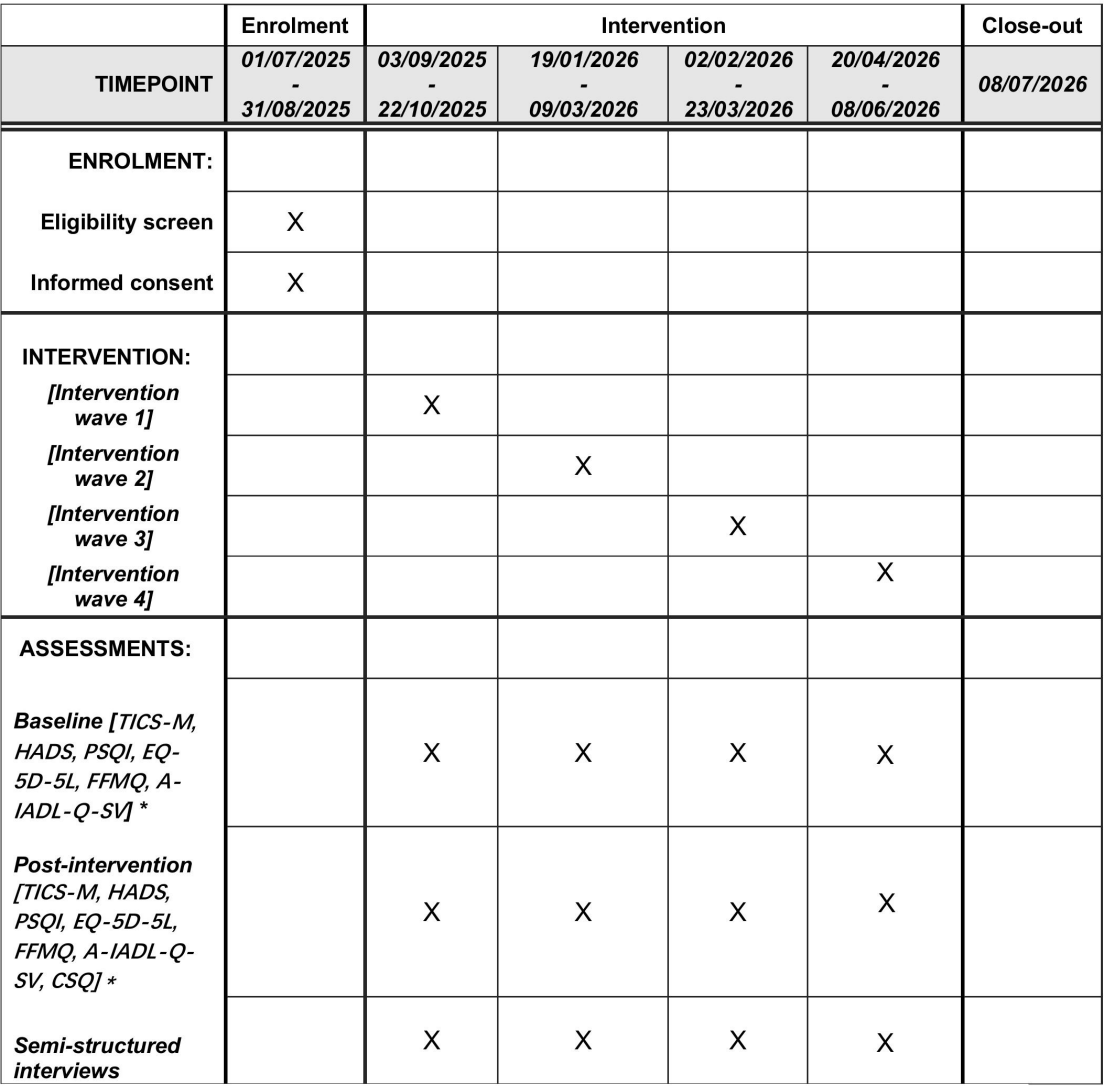
Study timeline Abbreviation: TICS-M, Modified Telephone Interview for Cognitive Status; HADS, Hospital Anxiety and Depression Scale; PSQI, Pittsburgh Sleep Quality Index; EQ-5D-5L, 5-level EQ-5D version; A-IADL-Q-SV, Amsterdam IADL Questionnaire – Short Version; FFMQ, Five-Facet Mindfulness Questionnaire; CSQ, Client Satisfaction Questionnaire. *Baseline assessments will be conducted one month before intervention; post-intervention assessments will be conducted within one month after intervention.

The project has started on 01/07/2025 and will end on 08/07/2026. Recruitment will continue until participants for the final wave have been confirmed, and data collection will be completed by 08/07/2026. The results are expected to be presented in June 2027. Recruitment has begun; a detailed timeline is shown in **Fig 1**.

## 4 Discussion

To the best of our knowledge, this is the first study to explore a live, online-delivered mindfulness program for older adults with MCI and dementia. Therefore, rather than conducting a large randomised controlled trial, we begin with a feasibility study to evaluate the program’s practicality and potential effectiveness.

Although previous studies have investigated online mindfulness meditation programs for older adults, they have not specifically targeted individuals with MCI or dementia. These programs were also delivered through various formats, including live web-based videoconferencing [56,57], self-learning via websites [58,59], iPod [60], and mobile applications [60–62]. Across different modalities, these studies have generally reported feasibility and acceptability. For instance, Minjung et al. [57] conducted a 12-week, group-based, online mindfulness-based dance/movement therapy via zoom. They recruited 16 older adults with age-related cognitive decline, and over 75% of participants completed more than nine sessions. Similarly, Helane [59] recruited 16 participants (eight in the mindfulness intervention group), and their six-week web-based self-learning mindfulness program achieved a 76% completion rate. Stav et al. [63] conducted a study with 82 community-dwelling older adults (intervention group: n = 64) in small groups (5–7 participants), delivering seven sessions (1–1.5 hours each) over 3.5 weeks, resulting in an 80.8% retention rate. The study design of Galluzzi et al. [56] is similar to ours, featuring eight-week live online sessions led by a meditation teacher, supplemented with recorded materials for home practice. Their study [56] found 66% of older adults completed the eight-week course, with significant improvements in verbal memory, attention, and executive functions.

Overall, these studies demonstrate that older adults report high satisfaction with online mindfulness programs, which improve their well-being and reduce loneliness and depression. Therefore, this suggests that online mindfulness programs might also benefit people with MCI or dementia.

### 4.1 Considerations for social interaction in group interventions

For group interventions, it is also important to consider the effects of social interaction. Previous studies have highlighted social connectedness as a key component of mindfulness-based interventions [63]. Group interactions, particularly in an eight-week structured program, contribute to overall benefits; however, it remains challenging to isolate the effects of mindfulness practice from those of social engagement. In our intervention, each session includes at least 20 minutes of group discussion, during which participants share experiences and personal stories, therefore fostering social support—an essential factor in reducing loneliness [64]. Therefore, establishing a control group in future studies would be beneficial to compare the effects of individual or self-guided interventions with those of group interventions, allowing for a deeper understanding of the distinct contributions of mindfulness meditation and social interaction.

### 4.2 Addressing technological literacy and accessibility

Older adults’ technology literacy is a key determinant of feasibility in this study. While previous studies have addressed this challenge by sending research assistants to participants’ homes to facilitate consent procedures and assessments [62], our study will complete these processes remotely via telephone or online platforms. This approach allows us to evaluate the feasibility of a fully remote process, which may enhance scalability and reduce travel burdens for both participants and researchers. Additionally, this design better simulates real-world digital health interventions, where clinicians are unlikely to provide in-home technological support.

To mitigate potential challenges, participants will receive instructional support through pre-recorded video tutorials and PDF guides. A videoconference option will also be available for participants who require assistance with understanding questionnaires or using Qualtrics for assessments. The ability of participants to navigate these digital tools independently or with the assistance of caregivers will provide valuable insights into the real-world applicability of remote mindfulness interventions for older adults with MCI and dementia.

### 4.3 Engagement strategies and follow-up

Maintaining participant engagement throughout the program is essential. Stav et al. [63] implemented a WhatsApp group to facilitate ongoing communication between participants and moderators and to share supplementary materials, including text, video, and audio files. In our study, we will adopt a similar strategy by establishing a WhatsApp group and conducting individual weekly calls to check participants’ experiences, address their difficulties in completing the course, and provide necessary support, thereby increasing adherence and program completion rates.

### 4.4 Descriptive focus for statistical analysis

Feasibility studies are conducted before a main study to answer “can this study be done?” [65] They are used to explore key parameters, such as participants’ willingness to join, number of eligible participants, follow-up rates, and adherence/compliance rates. Therefore, in this feasibility study, we focused on descriptive analysis rather than hypothesis testing. As recommended in guidelines for feasibility studies [66], these studies should not be used to estimate effect sizes, perform exploratory analyses of efficacy, or provide power calculations for statistical tests. The emphasis of feasibility study has shifted from informing sampling decisions to estimating confidence intervals (CIs); however, CIs remain wide for small sample size. Addressable questions are whether the study protocol is feasible, and whether participant retention and adherence are achieved.

Regarding missing data, it is less important to “deal with” them in a feasibility study and more important to understand why they are missing and what could be done in future definitive trials [67]. Therefore, we will describe the extent of missing data and record participants’ reasons for dropping out.

### 4.5 Limitations and future directions

One limitation of this study is that participants will be recruited mainly from memory clinics in London, which may reduce the generalizability of findings to rural areas with lower digital accessibility. Second, as the primary aim is to assess feasibility and acceptability, we did not plan a longer follow-up or a control group. Third, descriptive statistics do not allow for formal hypothesis testing or control for confounding factors. As such, any observed changes should be interpreted as preliminary and illustrative only, rather than as evidence of efficacy. Future studies should incorporate a larger randomized controlled study, with a more geographically and socioeconomically diverse sample to enhance external validity. In addition, a mid-point efficacy assessment and a longer follow-up should be included to evaluate both short-term and long-term efficacy.

Digital mindfulness programs offer some advantages over traditional face-to-face interventions, including improved standardization, increased accessibility, and greater personalization [68]. However, challenges remain, such as poor Wi-Fi connectivity, limited technological literacy, and insufficient technical support for older adults. This study will contribute to the growing body of evidence on digital mindfulness interventions, shedding light on how older adults, particularly those with cognitive impairments, can adapt to internet-based tools. Findings from this feasibility study will inform the design of larger RCTs and help refine digital mindfulness interventions to improve accessibility, engagement, and overall effectiveness in this population.

## Supporting information

None.
